# Cefazolin surgical prophylaxis in obesity: a body composition-driven population pharmacokinetic approach

**DOI:** 10.1128/aac.01677-25

**Published:** 2026-05-29

**Authors:** Andrew Wassef, Katarzyna Kosicka-Noworzyń, Hyunmoon Back, Anna Siemiątkowska, Christine Yohn, Ragui Sadek, Leonid Kagan, Luigi Brunetti

**Affiliations:** 1 Department of Pharmaceutics, Ernest Mario School of Pharmacy, Rutgers, The State University of New Jersey 15484 , Piscataway, New Jersey, USA; 2 Center of Excellence in Pharmaceutical Translational Research and Education, Ernest Mario School of Pharmacy, Rutgers, The State University of New Jersey 242612, Piscataway, New Jersey, USA; 3 Advanced Surgical and Bariatrics of New Jersey, Somerset, New Jersey, USA; 4 Department of General Surgery, Rutgers Robert Wood Johnson Medical School 12287 , New Brunswick, New Jersey, USA; 5 Department of Physical Pharmacy and Pharmacokinetics, Poznan University of Medical Sciences 37807, Poznań, Poland; 6 Center of Excellence for Metabolic and Bariatric Surgery, Robert Wood Johnson Barnabas University Hospital 25044, New Brunswick, New Jersey, USA; 7 Department of Pharmacy Practice, Rutgers, The State University of New Jersey 242612, Piscataway, New Jersey, USA; Providence Portland Medical Center, Portland, Oregon, USA

**Keywords:** cefazolin, antibiotics, surgical prophylaxis, obesity, pharmacokinetics, body composition, population pharmacokinetic modeling

## Abstract

Cefazolin serves as the antibiotic choice for surgical prophylaxis to prevent surgical site infection, yet consensus on dosing guidelines for patients with obesity is not well defined. To quantify cefazolin exposure in plasma and subcutaneous adipose tissue during abdominal surgery, and to build a popPK model comparing standard anthropometrics, calculated body-composition metrics, and directly measured body-composition values as predictors of interindividual variability. A single center prospective study was conducted at Robert Wood Johnson Barnabas University Hospital, NJ, USA. Institutional standard for dosing was 2 g < 120 kg, or 3 g of cefazolin for subjects ≥ 120 kg for 101 adult subjects. Serial blood samples and subcutaneous adipose tissue samples were collected for the assessment of cefazolin concentration. All subjects received body composition assessments. Cefazolin concentrations in plasma remained within the therapeutic range in all stratification groups, yet subcutaneous adipose tissue concentrations failed to meet therapeutic cut offs, with 2- to 8.5-fold decrease in target attainment in subjects with increasing body fat percentages and BMI. A three-compartment biophase model best characterized our data and served as our base popPK model. Stepwise covariate modeling of measured body composition metrics outperformed both calculated body composition metrics and standard anthropomorphic values. The results support the claim that existing weight-based dosing methods fall short at the site of action, while simultaneously providing a proof of concept for the first in-human body composition informed popPK model. Measured body composition metrics can enhance characterization of cefazolin prophylactic exposure for subjects with obesity receiving abdominal surgery.

## INTRODUCTION

Antibiotic surgical prophylaxis is the standard of care for the prevention of surgical site infections worldwide (SSI) (1–6). SSIs account for nearly 20% of all hospital acquired infections and are associated with a 2- to 11- fold increase in risk of infection-related mortality (7). SSI-related healthcare costs currently represent the highest financial burden with respect to infection in the United States. Approximately 160,000–300,000 patients develop SSIs annually in the USA alone, with an incidence of up to 45% for particular surgical procedures at increased risk of bacterial exposure (8–10). The burden of infection imposes nearly 10 billion in annual costs with heightened risk of hospital readmission, extended hospitalization, and nearly doubling the cost of treatment (11). The likelihood of SSI increases significantly among individuals with obesity. As body mass index (BMI) rises beyond 35, the chances of developing postoperative infections doubles (12–14). Obesity-driven factors such as altered pharmacokinetics (PK) and insufficient antibiotic penetration into subcutaneous tissue may exacerbate SSI risk, underscoring the need for refined perioperative antibiotic dosing strategies (15–19).

Cefazolin, a first-generation cephalosporin, remains the most frequently administered antibiotic for surgical prophylaxis worldwide (1–3, 5). As for all beta-lactams, the pharmacodynamic (PD) target is an antibiotic concentration above the minimum inhibitory concentration (MIC) of the expected pathogens for at least 40%–60% of the dosing interval. For surgical prophylaxis, it is desirable to achieve this PD target for the entire duration of the procedure (100%) (20). Current cefazolin surgical prophylaxis practice guidelines suggest weight-based dosing, with a dose adjustment for patients 120 kg and above (3 g vs 2 g), yet these guidelines do not address the complex relationships between body fat distribution and altered PK present in obesity (1–4, 17). Research suggests that conventional descriptors, such as total body weight (TBW), lean body weight (LBW), adjusted body weight (AdjBW), ideal body weight (IBW), or body mass index (BMI), do not adequately capture the variability in PK among patients with obesity (17, 21, 22). The exclusive use of these values for clinical dosing directions may not achieve therapeutic cefazolin tissue concentrations at the surgical site, leading to inadequate antimicrobial coverage, a known driver of SSI risk (23–26).

Population pharmacokinetic (PopPK) modeling can enhance antibiotic dosing precision by incorporating individual patient variables such as age, sex, and renal function. Existing PopPK models of cefazolin primarily examine therapeutic plasma exposure levels rather than tissue concentrations relative to MIC thresholds (27–29). Of the few studies investigating cefazolin penetration into subcutaneous adipose tissue, there is considerable interindividual variability which could not be explained by TBW, LBW, BMI, or other anthropometric measures (23, 30–32). Advances in quantitative imaging techniques, such as analytic morphomics (28), have shed light on the potential utility of more precise metrics of adiposity and its use in cefazolin dose refinement (33). Still, routine implementation of CT-based body composition analysis is limited and costly and, therefore, not universally feasible.

Body impedance analysis (BIA) provides a cost-effective noninvasive alternative to CT-based assessments by delivering detailed body composition measurements that surpass standard anthropometric measures (34, 35). The ability of BIA to accurately characterize body composition parameters like muscle-mass, fat-mass, and fat-free mass (34, 36) makes it especially helpful for measuring values which may prove to be significant factors affecting cefazolin exposure. We propose incorporating BIA-derived metrics into PopPK models to enhance prediction accuracy for subcutaneous tissue drug levels, in an effort to enable more precise dosing strategies for morbidly obese patients (BMI ≥ 30 kg/m²). This approach has the potential to address longstanding gaps in perioperative antibiotic surgical prophylaxis guidelines which would lead to better patient outcomes by reducing despairingly high incidence of SSIs in obese patients.

The principal objectives of this study are twofold. First, we aim to evaluate whether standard intravenous cefazolin prophylactic doses (2 g TBW < 120 kg, 3 g TBW ≥ 120 kg) achieve comparable exposure in patients undergoing abdominal surgery. Second, we seek to develop a robust PopPK model to comparatively evaluate standard available anthropometric values, calculated body composition metrics, and directly measured body composition values, to identify the optimal approach for describing interindividual variability of cefazolin exposure. The central hypothesis posits that patients with obesity who are administered institutional standard prophylactic cefazolin dosages will fail to attain similar antibiotic exposure, consequently placing them at heightened risk for SSI. Furthermore, we hypothesize that subcutaneous antibiotic exposure of obese individuals is significantly lower compared to non-obese counterparts receiving standard prophylactic regimens.

## MATERIALS AND METHODS

### Study design

This prospective, non-interventional study enrolled patients (age 18 to 65 years) undergoing sleeve gastrectomy and laparoscopic abdominal surgeries (i.e., hiatal, paraesophageal, ventral, umbilical, inguinal, incisional hernia repair). Patients who received cefazolin (participation in the study did not alter the selected drug or dose level) were included. Patients weighing < 120 kg received 2 g, while others weighing ≥ 120 kg received 3 g of cefazolin as an intravenous bolus every 4 h for the duration of the surgical period.

### Patient population

**Fig 1:**
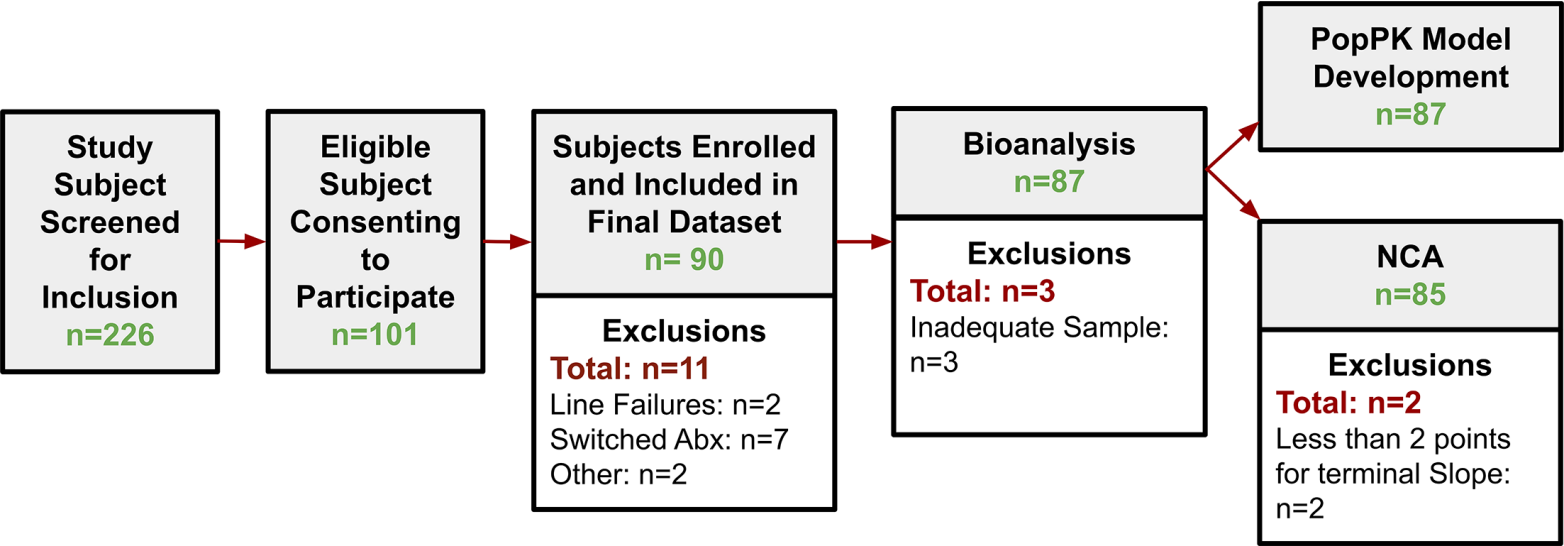
Enrollment Flow Chart.

All patients receiving sleeve gastrectomy (BMI > 30 kg/m^2^ with comorbidities or BMI > 35 kg/m^2^ is required for bariatric surgery) or laparoscopic abdominal surgeries (BMI < 30 kg/m²) were screened and approached for study participation. Patients with liver impairment (elevations in aspartate aminotransferase [normal range, 10–55 units/L] or alanine aminotransferase [normal range 10–50 units/L]) greater than three times the upper limit of normal, moderate to severe renal dysfunction (creatinine clearance < 50 mL/min), or those with a documented penicillin or cephalosporin allergy were excluded. We enrolled 101 subjects (*n*= 71 gastrectomy and *n*= 30 laparoscopic abdominal surgeries). Seven participants who received alternative antibiotics during surgery and two subjects who experienced line failures were excluded from the study (Fig. 1). After exclusions, all subjects received one or two doses of intravenous cefazolin during the surgical period (*n* = 85 one dose, *n* = 2 two doses).

### Sample collection

Serial blood samples for all subjects were drawn from a separate antecubital line following intravenous cefazolin bolus dose (2 g or 3 g) to prevent line contamination. Blood samples were collected according to standard methods, with a discard volume of 2 mL to clear catheter dead space and a 5 mL flush of saline between samples. At each designated sampling time from drug administration (baseline, 5 min, 30 min, 60 min, 120 min, and 240 min), permitting deviations to minimize interference with operative care. Four milliliters of venous blood was collected, placed on ice, and subsequently centrifuged at 3,000 rpm. The plasma fraction was separated into aliquots and frozen at −80°C for later cefazolin concentration analysis. Two subcutaneous abdominal adipose tissue samples (approximately 1 g) were removed upon surgical incision and surgical closure at laparoscopic port sites, with time recorded post cefazolin dose. Collection was performed with a surgical blade and forceps to avoid electrocautery of the tissue specimen and potential degradation of cefazolin due to extreme heat. Tissue specimens were quickly transferred on ice and stored at −80°C until further analysis.

### Determination of body composition and patient variables

Similar to Brunetti and colleagues (37), we performed segmental BIA on all participants using the Tanita MC-780 (Tanita Corporation, Arlington Heights, IL, USA) body composition analyzer (37). Body composition was collected for all subjects in accordance with manufacturer instructions and was repeated to ensure good reproducibility between analyses. Mean body composition values were subsequently used for further analysis. Output values included total body weight (TBW), body fat mass (FM), fat-free mass (FFM), muscle mass, bone mass, total body water (WTRAMT), extracellular water (ECW), intracellular water (ICW), and basal metabolic rate (BMR) among other values (38). Values were provided as a total mass or percentage of total body weight and were available for segmental analysis (right and arm, right and left leg, and abdominal trunk). Other calculated measures of body composition such as lean body weight (LBW), ideal body weight (IBW), and adjusted body weight (AdjBW) were calculated based on standard equations (39, 40). FM was calculated by subtracting LBW value of each subsequent equation (Jamanthesian [41], James [42], Hume [43], Boer [44] equations) from subjects TBW. Finally, creatinine clearance (CrCl) was calculated using the Cockcroft-Gault equation, and estimated glomerular filtration rate (eGFR) was calculated using the CDK-EPI equation (45–47). Subject demographic information (including age, sex, height, race, and ethnicity), as well as comorbid conditions (such as hyperlipidemia, hypertension, diabetes mellitus, nonalcoholic fatty liver disease, obstructive sleep apnea, and metabolic syndrome), and laboratory measurements (including albumin, bilirubin, aspartate aminotransferase, alanine aminotransferase, blood urea nitrogen, serum creatinine, total protein, and platelet count) were extracted from the subjects’ medical records.

### Chemicals

Cefazolin sodium and cloxacillin sodium (both from Alfa Aesar), as well as LCMS grade solvents and additives, were purchased from Fisher Scientific (Fair Lawn, NJ, USA), piperacillin sodium—from Sigma-Aldrich (St. Louis, MO, USA), a phosphate-buffered saline (PBS)—from Corning (Corning, NY, USA), zirconium oxide beads for tissue homogenization—from Next Advance (Troy, NY, USA), Captiva EMR-lipid plates for lipids removal—from Agilent Technologies (Santa Clara, CA, USA), PVDF filters—from Fisher Scientific (Fair Lawn, NJ, USA). Blank subcutaneous rat adipose tissue, utilized for calibration curves in mass spectrometric analyses, was collected at Rutgers University from adult male Sprague Dawley rats (protocol no. ID999900460, approved by the Rutgers IACUC). Blank human plasma (K_2_EDTA), utilized for calibration curves in HPLC-UV analyses, was purchased from BioIVT (Westbury, NY, USA).

### Determination of cefazolin in adipose tissue

Total subcutaneous adipose tissue concentration of cefazolin was determined with a validated LC-MS/MS method (48). Briefly, the Sciex QTRAP 6500+ mass spectrometer coupled with the ExionLC AD system was used for quantitation, and the instrument was operated in MRM mode. The elution was achieved under gradient conditions on the Phenomenex Synergi Fusion-RP column (50 × 2 mm, 4 μm) with 5 mM ammonium formate in 0.1% formic acid in water (vol/vol) and 0.1% formic acid in acetonitrile (vol/vol). The flow rate was 0.3 mL/min, and the column and autosampler temperatures were 40°C and 10°C, respectively. The fat tissue samples were homogenized with PBS and thoroughly cleaned before mass spectrometric analysis. Proteins were removed by precipitation (0.5% formic acid in acetonitrile), while lipids—through utilizing the Agilent Captiva EMR-Lipid plates. The method’s calibration range was 0.15–150 μg/g in adipose tissue. Cloxacillin was used as an internal standard.

### Determination of cefazolin in plasma

Total plasma concentrations of cefazolin were measured with a validated HPLC-UV method. Specifically, Agilent 1260 HPLC system equipped with a DAD detector was used. Separation was achieved with a Phenomenex Kinetex XB-C18 column (150 × 2.1 mm, 2.6 µm) and a gradient flow of 0.1% formic acid in water (vol/vol; mobile phase A), 0.1% formic acid in acetonitrile (vol/vol; mobile phase B): methanol (mobile phase C). The flow rate was 0.3 mL/min, with the gradient conditions: 0 min—92:5:3 (mobile phase A:B:C), 15 min—62:35:3, 16.5 min—62:35:3, 18 min—92:5:3. The detection wavelength was 270 nm, and the column and autosampler temperatures were 45°C and 10°C, respectively. Piperacillin (75.0 µg/mL in acetonitrile) was used as an internal standard (retention time was 15.7 min). Cefazolin retention time was 8.5 min, and limit of quantification (LOQ) was 0.5 µg/mL. Plasma samples (200 µL) were spiked with 200 µL water (or aqueous standards of cefazolin for calibration) and vortexed. Then, 200 µL of internal standard solution and 500 µL of pure acetonitrile were added for protein precipitation. Samples were centrifuged for 2 min at 15,700 × *g*. Supernatant (1,000–1,100 µL) was transferred into a glass vial, and 2 mL of dichloromethane was added for extraction. Samples were shaken for 5 min and centrifuged for 10 min at 1,902 × *g* at 4°C. The upper (aqueous) layer was transferred into the PVDF filters, and the samples were centrifuged for 2 min at 13,000 × *g*. The 30 µL of the filtrate was injected to the HPLC column. The method’s calibration range was 0.5–250 µg/mL. Precision and accuracy of the method were 0.6% –13.7% and 96.4%–105.8%, respectively. Dilution integrity was confirmed, and plasma samples with cefazolin concentration >250 µg/mL were analyzed after dilution with PBS (1:1, vol/vol). The method was checked for selectivity. Piperacillin interfered with penicillin G, and cefazolin with ceftriaxone; no interferences were found for cefoxitin, cefotetan, tazobactam, bupivacaine, ondansetron.

### Data analysis

Demographic characteristics were summarized as counts and percentages within every specified comparison group. Body-composition variables were grouped by dose level (2 g vs 3 g) summarized as mean ± standard deviation (SD) and, in parallel, as median (range) to characterize distributional spread. Non- compartmental analysis (NCA) parameters were likewise expressed as mean ± SD within each group and compared with two-sample *t*-tests when two groups were present or one-way ANOVA with Bonferroni correction when more than two groups were present; a two-sided *P*-value ≤ 0.05 was interpreted as statistically significant. Target attainment of cefazolin MIC (16, 8, or 4µg/mL) was reported as the count and percentage of participants above the target in each group and analyzed with fisher exact test where a Holm-Bonferroni *P*-value ≤ 0.05 was interpreted as statistically significant. An odds ratio test (OR) for target attainment was also performed where an OR ≥ 2.0 was considered an indication of a potentially meaningful effect, even if statistical significance (*P* < 0.05) was not achieved. All analyses were stratified a priori by (i) dose (2 g vs 3 g), (ii) BMI category according to the World Health Organization classification (49) (normal weight 18.5-24.9 kg/m², overweight 25.0-29.9 kg/m², class I obesity 30.0–34.9 kg/m², class II obesity 35.0–39.9 kg/m^2^, and class III obesity ≥ 40  kg /m²), and (iii) sex-specific percent body fat thresholds distinguishing healthy from unhealthy subjects (unhealthy >30 male, >35 female) (50). To preserve comparable sample sizes across BMI strata for NCA, BMI categories were collapsed into three broader groups (18.5–29.9, 30.0–39.9, and ≥ 40). For subjects with BMI ≥ 40, NCA parameters were further evaluated by dose (2 g vs 3 g) to explore dose-related differences within a similar body composition range. All statistical analyses were conducted using GraphPad Prism (version 10, GraphPad Software, San Diego, CA; 2023 release).

### Non-compartmental analysis (plasma)

Total cefazolin concentrations in plasma were analyzed by noncompartmental analysis using Pkanalix software (Version 2024R1; Lixoft SAS, Antony, France). The area under the concentration-time profile for the sampling interval (AUC_0→τ_), total area under the concentration-time profile (AUC_0→inf_), half-life (*t*_1/2_), clearance (CL), and volume of distribution (*V*_d_), volume at steady state (*V*_ss_) were calculated. The AUC was calculated using the linear-log trapezoidal method. The *t*_1/2_ was calculated as ln(2)/*λ*_z_, where *λ*_z_ is the terminal elimination rate constant. The terminal elimination rate constant was estimated by linear regression analysis of the terminal portion of the concentration-time profile utilizing at minimum two points in terminal slope. Clearance was calculated as dose/AUC_0→inf_. The volume of distribution was calculated as dose/(*λ*_z_ × AUC_0→τ_). Additionally, AUC was normalized according to total dose (AUC_0→inf/Dose_) and dose per kg (AUC_0→inf/(Dose/kg)_) to determine dose and weight-dependent variability.

### Compartmental analysis

#### Model development

Total cefazolin concentrations in plasma and SC adipose tissue were analyzed by compartmental analysis using Monolix software (Version 2024R1; Lixoft SAS, Antony, France). The modeling process was conducted in three major steps. First, candidate structural models were tested using only the plasma concentration time data. One-, two-, and three-compartment models were evaluated alongside different residual error models (additive, proportional, constant, or combined 1 or 2) under both normal and log-normal assumptions. The model that best characterized the plasma data based on goodness-of-fit plots, parameter plausibility, objective function value (OFV), and parameter estimates was retained for further expansion.

Second, plasma and subcutaneous adipose tissue concentrations were co-modeled to capture the interplay between these compartments. Various parameterizations for the plasma to subcutaneous transfer were explored, including (i) assessment whether any of the peripheral compartments from the model based on plasma data alone accurately represent subcutaneous (SC) tissues; (ii) additional peripheral SC tissue compartment to the systemic disposition model (with additional distribution rate constant); (iii) capturing SC tissue concentration as proportional to plasma (using a partition coefficient); (iv) a biophase model (with rate constant and a partition coefficient). Model structural configuration was chosen based on model diagnostics, improved statistical criteria, and physiologic plausibility. Each model was assessed based on statistical fit, plausibility of parameter estimates, relative standard errors (RSE), and diagnostic plots (observed vs predicted concentrations, weighted residuals).

#### Covariate evaluation

After establishing the base structural model for systemic disposition and subcutaneous adipose tissue distribution, demographic and clinical variables (Fig. 5: group 1) calculated values of body composition (Fig. 5: group 2), and measured values of body composition (Fig. 5: group 3) were independently tested as potential covariates on various pharmacokinetic parameters. These variables were first screened for physiological relevance to the respective parameter open for selection. Continuous covariates were centered on their median values, and categorical covariates were parameterized using indicator variables. Variables were encoded as binary indicators (0 = absence, 1 = presence), where a value of 1 signified either the presence of the disease state or female sex, depending on the covariate in question. Multiple covariates for the same parameters were tested in a multiplicative fashion.

The influence of covariates on individual pharmacokinetic parameters was modeled according to the following equations:

#### Continuous normal distribution of parameters


$$P_{i}=P_{pop}{\left(\frac{COV_{i}}{COV_{med}}\right)}^{\beta_{cont}}exp(\eta_{P,i})$$

#### Continuous LogNormal distribution of parameters


$$\log P_{i}=\log P_{\text{pop}}+\beta_{\text{cont}}\left(\log COV_{i}-\log COV_{\text{med}}\right)+\eta_{P,i}$$

Here *P*_pop_ is the typical population value, *β*_cont_ is the fixed covariate coefficient estimated by SAEM, and *η*_*P*,_*_i_* captures the remaining inter‑individual variability (IIV).

#### Categorical normal distribution of parameters


$$P_{i}=P_{\text{pop}}\left(1+\theta_{\text{cat}}\;CAT_{i}\right)\exp\left(\eta_{P,i}\right),\;\theta_{\text{cat}}=\exp(\beta_{\text{cat}})-1$$

#### Categorical LogNormal distribution of parameters


$$\log P_{i}=\log P_{\text{pop}}+\beta_{\text{cat}}\;CAT_{i}+\eta_{P,i}$$

Here, *P*_pop_ applies to the reference category (indicator = 0) and *P*_pop_(1 + *θ*_cat_) to the non-reference category (indicator = 1).

A stepwise covariate modeling approach was then applied: forward inclusion at *P* < 0.05, followed by backward elimination at *P* < 0.01, with maximum likelihood ratio tests guiding model improvements. A drop in the OFV by at least 6.63, after forward inclusion and backward elimination, was required to confirm a covariate’s significance for final inclusion on our base model. All SCM was performed in Monolix (Version 2024R1; Lixoft SAS, Antony, France).

### Bootstrap

Non-parametric bootstrap resampling was performed to quantify the precision of all fixed and random effect parameters. 1,000 replicate data sets were generated by resampling subjects with all of their observations with replacement from the original analysis file. Each replicate was re-estimated with the final PopPK model using the original run’s parameter estimates as starting values and identical SAEM settings; computations were executed in parallel on five CPU cores. Replicates were discarded if SAEM failed to converge. If any relative standard error exceeded 100%, or if the OFV was >30 units above the median of successful runs, it was discarded. For every parameter, the bootstrap distribution was summarized by its median, the 2.5th and 97.5th percentiles (percentile-based 95% confidence interval), and the bootstrap relative standard error (βRSE = SD / median × 100%). These empirical intervals do not rely on asymptotic Fisher information and were reported as the primary measure of parameter uncertainty. Bootstrapping was performed in Monolix (Version 2024R1; Lixoft SAS, Antony, France).

## RESULTS

### Subject characteristics

Total enrollment of subjects was 101, and data from 87 subjects were available for final analysis (Fig. 1). Clinical and demographic data were summarized and stratified according to dose, BMI, and fat percentage. Of the 87 subjects, 46 (53%) were females, and 66 (76%) were white. Subjects in 2 vs 3 g dose group had an average [mean, SD] age of [49, 13.4] and [41, 14.6] and mean weight of [34.8, 10.1] and [43.9, 6.9], respectively. Subjects in 2 vs 3 g groups had mean FM of [36.1, 16.3] and [60.4, 18.8] and mean FFM of [59.1, 16.32] and [70.8, 16.18]. Select measures of body composition are shown in Table 1 with expanded measured and calculated values of body composition in Table S1.

**TABLE 1:** Comparison of Basic Demographics Between Groups of Interest^*a*^

Characteristics	Dosage		BMI (kg/m^2^)					Fat percentage (%)	
	2 g (if <120 kg)	3 g (if ≥ 120 kg^*b*^)	Healthy 18.5–24.9	Overweight 25–29.9	Obese I 30–34.9	Obese II 35–39.9	Obese III ≥40	Healthy^*c*^	Unhealthy^*d*^
**Demographics**	***N*** **(%)**								
Sample size	57	30	10	8	16	17	36	17	70
Female	31 (54.4)	15 (50)	6 (60)	1 (12.5)	5 (31.3)	10 (58.5)	24 (66.7)	9 (41.2)	37 (55.7)
White	44 (77.2)	22 (73.3)	7 (70)	6 (75)	14 (87.5)	15 (88.2)	24 (66.7)	12 (70.6)	54 (77.1)
African American	5 (8.8)	6 (20)	2 (20)	0	0	1 (5.9)	8 (22.2)	2 (11.8)	9 (12.9)
Asian	3 (5.3)	0	1 (10)	1 (12.5)	0	0	1 (2.8)	2 (11.8)	1 (1.4)
Unknown	4 (7)	1 (3.3)	0	1 (12.5)	2 (12.5)	1 (5.9)	1 (2.8)	1 (5.9)	4 (5.7)
More than One Race	1 (1.8)	1 (3.3)	0	0	0	0	2 (5.6)	0	2 (2.9)
Bariatric surgery	29 (50.9)	28 (96.6)	0	0	7 (43.7)	16 (94.1)	35 (97.2)	0	58 (82.9)
	**Mean (SD)**								
Age (years)	49 (13.4)	41 (14.6)	57 (9)	61 (16.5)	50 (14.8)	45 (8.4)	39 (12.5)	57 (11.5)	44 (13.6)
Weight (kg)	95.41 (18.2)	146.9 (24.6)	69 (11)	89.8 (17.7)	99.6 (16.5)	109.5 (17.5)	137.5 (27.3)	76.8 (16.8)	121.6 (28.2
Height (cm)	170.1 (11.9)	176.7 (14.3)	172.4 (9.2)	178.7 (15.5)	174.9 (14.9)	171.7 (13)	169.9 (12.5)	174.5 (12.4)	171.7 (13.2)
**Body composition**	**Mean (SD)**								
Fat mass (kg)	36.1 (16.3)	60.4 (18.8)	16.4 (4.49)	20.6 (4.49)	35.2 (7.04)	46.6 (12.3)	60.4 (15.9)	17.76 (4.42)	50.8 (17.08)
Muscle mass (kg)	56.1 (15.56)	67.2 (15.44)	49.9 (11.1)	65.7 (16.4)	62.5 (16.4)	67.4 (14.98)	69.1 (16.1)	56.1 (15.56)	67.2 (15.44)
Fat free mass (kg)	59.1 (16.32)	70.8 (16.18)	52.6 (11.65)	69.2 (17.2)	65.8 (15.5)	70.95 (15.68)	72.7 (16.8)	59.06 (16.32)	70.76 (16.18)
Body water mass (kg)	41.4 (11.6)	50.7 (12.5)	36.7 (7.87)	48.5 (12.49	45.7 (10.47)	51.21 (11.84)	52.7 (13.5)	41.42 (11.68	50.74 (12.5)
**Labs**	**Mean (SD)**								
AST (IU/L)	31.7 (29.4)	26.5 (10.8)	23.7 (10.8)	29.9 (9.8)	41 (49.8)	34 (19.8)	24.7 (10)	26.8 (10.7)	30.6 (30)
ALT (IU/L)	41.3 (62.5)	40 (14.8)	21.2 (12.9)	31.3 (10.9)	43.2 (35.7)	66.1 (105.3)	29.4 (15)	26.5 (12.6)	41.2 (56.6)
Bilirubin (mg/dL)	0.52 (0.37)	0.43 (0.23)	0.5 (0.23)	0.85 (0.51)	0.37 (0.12)	0.66 (0.46)	0.37 (0.21)	0.62 (0.39)	0.45 (0.31)
Albumin (g/dL)	4.3 (0.46)	4.2 (0.27)	4.2 (0.19)	4.4 (0.25)	4.3 (0. 39)	4.4 (0.72)	4.2 (0.23)	4.29 (0.23)	4.26 (0.44)
CrCL (mL/min)	153.2 (61.4)	270.9 (88.9)	96.5 (31.2)	108.4 (24.3)	150.2 (54.2)	180.2 (41.3)	263.2 (85.3)	101.6 (29)	214.9 (86)
eGFR (mL/min/1.73^2^)	103.9 (17.02)	108.3 (19.8)	104.25 (14.4)	104.3 (13.9)	101.1 (17.5)	105.4 (13.8)	107.6 (21.5)	105.7 (12.8)	105.2 (19.02)
**Disease state**	***N*** **(%)**								
Dyslipidemia	18 (31.6)	11 (36.7)	3 (30)	3 (37.5)	6 (37.5)	7 (41.2)	10 (28.6)	6 (35.3)	17 (33.3)
HTN	27 (47.4)	13 (43.3)	3 (30)	7 (87.5)	9 (56.3)	8 (47.1)	13 (36.1)	9 (52.9)	31 (44.3)
T2DM	11 (19.3)	6 (20)	3 (30)	3 (37.5)	4 (25)	2 (11.8)	5 (13.9)	6 (35.3)	11 (15.7)
NASH	6 (10.5)	6 (20)	0	0	1 (6.3)	4 (23.5)	7 (19.4)	0	12 (17.1)
Metabolic syndrome	14 (24.6)	12 (40)	0	1 (12.5)	3 (18.8)	9 (52.9)	13 (36.1)	1 (5.9)	25 (35.7)
OSA	2 (36.8)	11 (36.7)	1 (10)	3 (37.5)	6 (37.5)	6 (35.3)	16 (45.7)	3 (17.6)	29 (42)

^*a*^ ALT, alanine transaminase; AST, aspartate aminotransferase; CrCl, creatinine clearance; eGFR, estimated Glomerular Filtration Rate; HTN, hypertension; NASH, non-alcoholic steatosis; OSA, obstructive sleep apnea; T2DM, type II diabetes mellitus.

^*b*^ One subject in the 3 g group weighed less than 120 kg (118 kg).

^*c*^ Healthy fat percentage characterized as total body fat percentage of <30% for males, <35% for females.

^*d*^ Unhealthy fat percentage characterized as total body fat percentage ≥30% for males, ≥35% for females.

### Noncompartmental analysis

**Fig 2:**
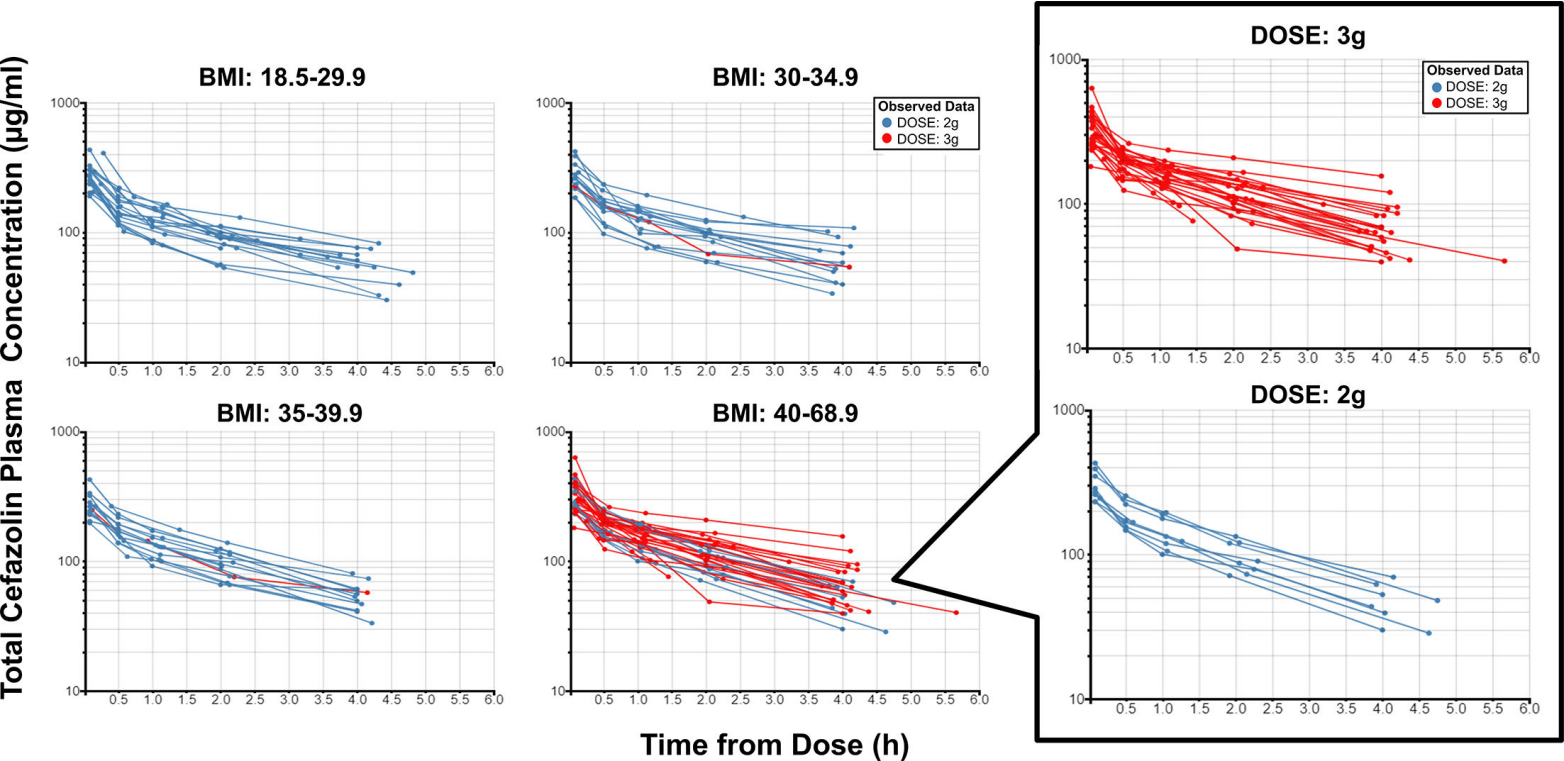
Individual total cefazolin plasma concentration time profiles after intravenous administration of 2 g or 3 g dose in subjects receiving abdominal surgery (*n* = 87).

PK data from 85 subjects were available for NCA analysis of plasma cefazolin exposure (Fig. 1). Figure 2 summarizes the plasma cefazolin concentration–time curves stratified by BMI and colored according to dose level. Cefazolin exposure was comparable across BMI groups, with slight differences only in participants with BMI > 40  kg/m^2^ who received a 3 g dose. Summary pharmacokinetic parameters stratified by dose, BMI, fat percentage, and dose for BMI >40 kg/m^2^ are presented in Table 2. Subjects receiving a 3 g cefazolin dose exhibited significantly higher CL 4.39 (L•h⁻¹) compared to the 2 g dose group 3.2 (L•h⁻¹). Conversely, the 2 g dose group had significantly higher dose normalized AUC_0→inf/Dose,_ 0.36 (h•L⁻¹) compared to the 3 g group 0.27 (h•L⁻¹). Additionally, subjects in the 3 g group demonstrated significantly greater *V*_d_ and *V*_ss_ 13.56 (L), 12.7 (L) compared to the 2 g group 11.07 (L), 10.3 (L). When grouped according to fat percentage, healthy subjects exhibited significantly lower dose per kilogram normalized AUC_0→inf/(Dose/kg),_ 23.56 (h•L⁻¹) as compared to their counterparts AUC_0→inf/(Dose/kg),_ 38.19 (h•L⁻¹). BMI alone did not yield significant pharmacokinetic differences, subjects with BMI >40 showed significant variations in *V*_ss_ with values of 8.59 (L) and 12.20 (L), as well as *V*_d._ with values of 9.44 (L) and 13.19 (L) when comparing 2 g vs 3 g dose groups.

**TABLE 2:** Comparison of basic pharmacokinetic parameters by non-compartmental analysis between groups of interest^*a*^

Characteristics	ALL	Dosage		BMI (kg/m^2^)			Fat percentage		BMI > 40 (kg/m^2^)	
		2 g (if < 120 kg)	3 g (if ≥ 120 kg)^*b*^	18.5–29.9	30–39.9	>40	Healthy^*c*^	Unhealthy^*d*^	2 g	3 g
N	85	56	29	18	32	35	15	70	10	25
**Parameters**	**Mean (SD)**									
AUC_0-inf_ (h∙g∙L^−1^)	0.74 (0.32)	0.72 (0.31)	0.8 (0.34)	0.66 (0.22)	0.77 (0.37)	0.77 (0.33)	0.61 (0.19)	0.77 (0.34)	0.63 (0.16)	0.83 (0.34)
AUC_0-inf/Dose_ (h∙L^−1^)	0.33 (0.15)	**0.36 (0.16)**	**0.27 (0.11)**	0.33 (0.11)	0.38 (0.19)	0.28 (0.11)	0.3 (0.097)	0.33 (0.16)	0.31 (0.08)	0.28 (0.12)
AUC0-_inf/(Dose/kg)_(h∙L^−1^)	35.61 (17.11)	34.34 (18.1)	38.08 (15.01)	25.61 (9.29)	38.71 (21.3)	37.92 (14.05)	**23.56 (7.62)**	**38.19 (17.1)**	35.46 (8.66)	39.66 (14.9)
AUC_0-t_ (h∙g∙L^−1^)	0.48 (0.22)	0.46 (0.24)	0.52 (0.16)	0.41 (0.12)	0.48 (0.3)	0.52 (0.15)	0.39 (0.13)	0.5 (0.15)	0.49 (0.12)	0.55 (0.13)
CL (L∙h⁻¹)	3.59 (1.47)	**3.2 (1.16)**	**4.35 (1.72)**	3.44 (1.42)	3.1 (1.1)	4.1 (1.65)	3.1 (1.42)	3.57 (1.49)	3.37 (0.85)	4.15 (1.58)
*T*_1/2_ (h)	2.58 (1.21)	2.66 (1.34)	2.44 (0.91)	2.71 (1.13)	2.83 (1.51)	2.29 (0.87)	2.52 (1.06)	2.6 (1.25)	1.97 (0.33)	2.46 (0.91)
*V*_ss_ (L)	11.12 (3.31)	**10.3 (3.34)**	**12.7 (2.67)**	11.03 (2.75)	10.8 (4.12)	11.44 (2.77)	11.15 (2.82)	11.11 (3.43)	**8.59 (1.90)**	**12.30 (2.27)**
*V*_d_ (L)	11.92 (3.53)	**11.07 (3.6)**	**13.56 (2.75)**	11.89 (3.15)	11.57 (4.32)	12.27 (2.9)	12.07 (3.3)	11.89 (3.59)	**9.44 (2.19)**	**13.19 (2.39)**

^*a*^ Cells highlighted in bold indicate significant *T*-test value with *P* < 0.05 within the respective stratification group.

^*b*^ One subject in the 3 g group weighed less than 120 kg (118 kg).

^*c*^ Healthy fat percentage characterized as total body fat percentage of <30% for males, <35% for females.

^*d*^ Unhealthy fat percentage characterized as total body fat percentage >30% for males, >35% for females.

**Fig 3:**
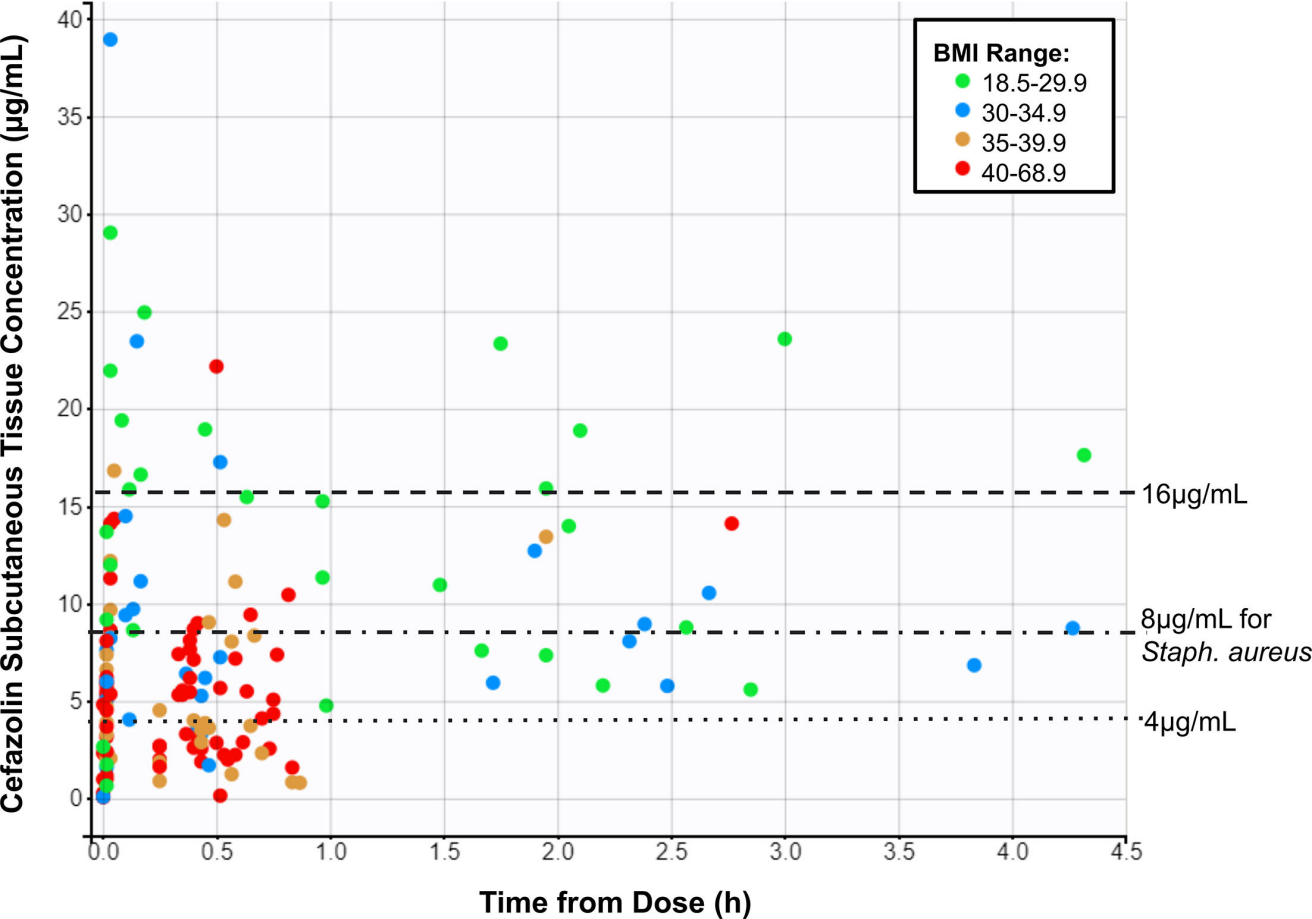
Individual total cefazolin subcutaneous adipose tissue concentration time profiles according to BMI strata after intravenous administration of 2 g or 3 g dose in subjects receiving abdominal surgery (*n* = 87).

Subcutaneous adipose cefazolin concentrations are depicted in Fig. 3 relative to several MIC targets thresholds. Time above MIC targets was assumed to be 100% for the duration of surgery in line with clinical surgical prophylaxis standards (3, 51, 52). Total MIC target tissue attainment for the entire duration of surgery is 3.4% at an MIC of 16, 17.2% at 8, and 40.2% at 4 (µg/mL) (Table 3). When stratified by BMI and body fat percentage, individuals with higher values demonstrated a proportionally 2- to 8.5-fold increased odds of not achieving tissue target concentrations, a pattern observed consistently across all MIC thresholds. Figure 3 further illustrates a statically significant inverse relationship between subcutaneous adipose cefazolin concentrations and BMI, notably highlighting that the majority of subtherapeutic concentrations occurred among subjects with BMI ranging from 35 to 68.9 at the MIC threshold of 4–8 (µg/mL).

**TABLE 3:** Subcutaneous adipose cefazolin concentration relative to MIC target attainment

Stratification		Subcutaneous adipose tissue collection time	Target attainment		
			>MIC (16 µg/mL)	>MIC (8 µg/mL)	>MIC (4 µg/mL)
		*N* (% within respective stratification group) OR [*P* value, 95% Confidence Interval]			
**ALL (*****N*** **= 87)**		Incision	11 (12.6)	30 (34.6)	47 (54)
		Closure	6 (6.9)	29 (33.3)	58 (66.6)
		Duration of surgery^*d*^	3 (3.4)	15 (17.2)	35 (40.2)
**DOSE (g)**	2 g < 120 (*N* = 57)	Duration of surgery^*d*^	2 (3.5)	11 (19.3)	24 (42.1)
	3 g ≥ 120^*a*^ (*N* = 30)		1 (3.3)	4 (13.3)	11 (36.7)
**BMI (kg/m** ^**2**^ **)**	BMI: 18.5–29.9 (*N* = 18)	Duration of surgery^*d*^	2 (11.1)	6 (33.3)	11 (61)
	BMI: 30–34.9 (*N* = 16)		0 (0)^*e*^	4 (25)	11 (69)
	BMI: 35–39.9 (*N* = 17)		1 (5.9)^*e*^	3 (17.6)^*e*^	3 (18) 7.3 [0.013, 1.5–35.1]^*f*^
	BMI: >40 (*N* = 36)		0 (0)^*e*^	2 (5.6) 8.5 [0.015, 1.5–48.0]^*f*^	10 (28) 4.1 [0.021, 1.2–13.5]^*f*^
**Fat percent (%)**	Healthy^*b*^ (*N* = 17)	Duration of surgery^*d*^	2 (11.8)	6 (35.3)	10 (58.8)
	Unhealthy^*c*^(*N* = 70)		1 (1.4)^*e*^	9 (12.9)^*e*^	25 (35.7)^*e*^

^*a*^ One subject in the 3 g group weighed less than 120 kg (118 kg).

^*b*^ Healthy fat percentage characterized as total body fat percentage of <30% for males, <35% for females.

^*c*^ Unhealthy fat percentage characterized as total body fat percentage >30% for males, >35% for females.

^*d*^ Duration of surgery is defined as time of operative incision to time of operative closure.

^*e*^ Indicate an odds ratio (OR) of ≥2-fold increase with the reference group being either 2 g, BMI: 18.5–29.9, or healthy fat % respectively.

^*f*^ Indicate an OR of ≥2-fold increase, and a significant Fisher-exact test value with *P *< 0.05 between groups, with the reference group being either 2 g, BMI: 18.5–29.9, or healthy fat %, respectively.

### PopPK modeling

**Fig 4:**
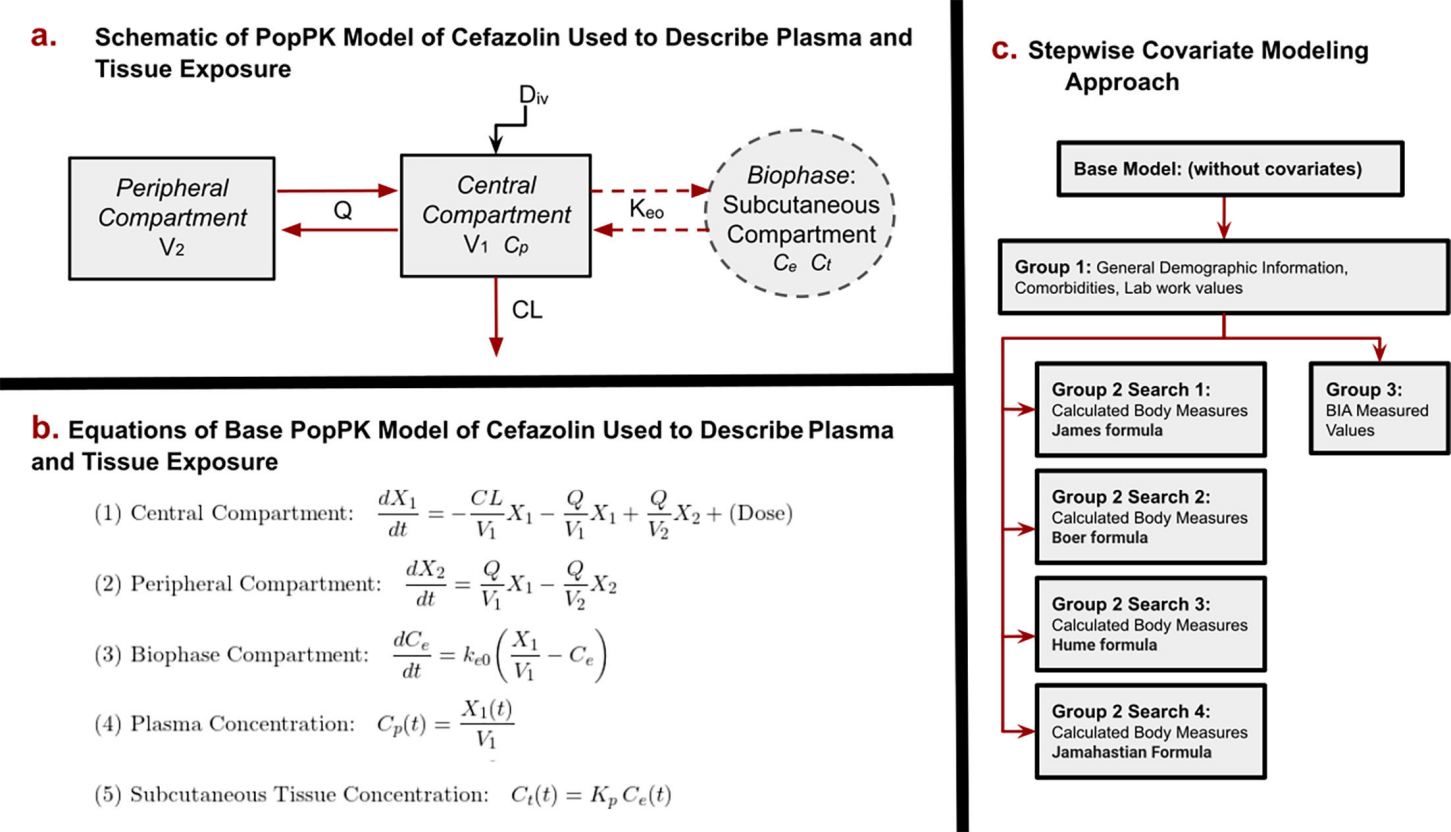
(**a**) Schematic of popPK Model of Cefazolin used to describe plasma and tissue exposure. (**b**) Equations of base popPK model of cefazolin used to describe plasma and tissue exposure. (**c**) Stepwise covariate modeling approach.

**Fig 5:**
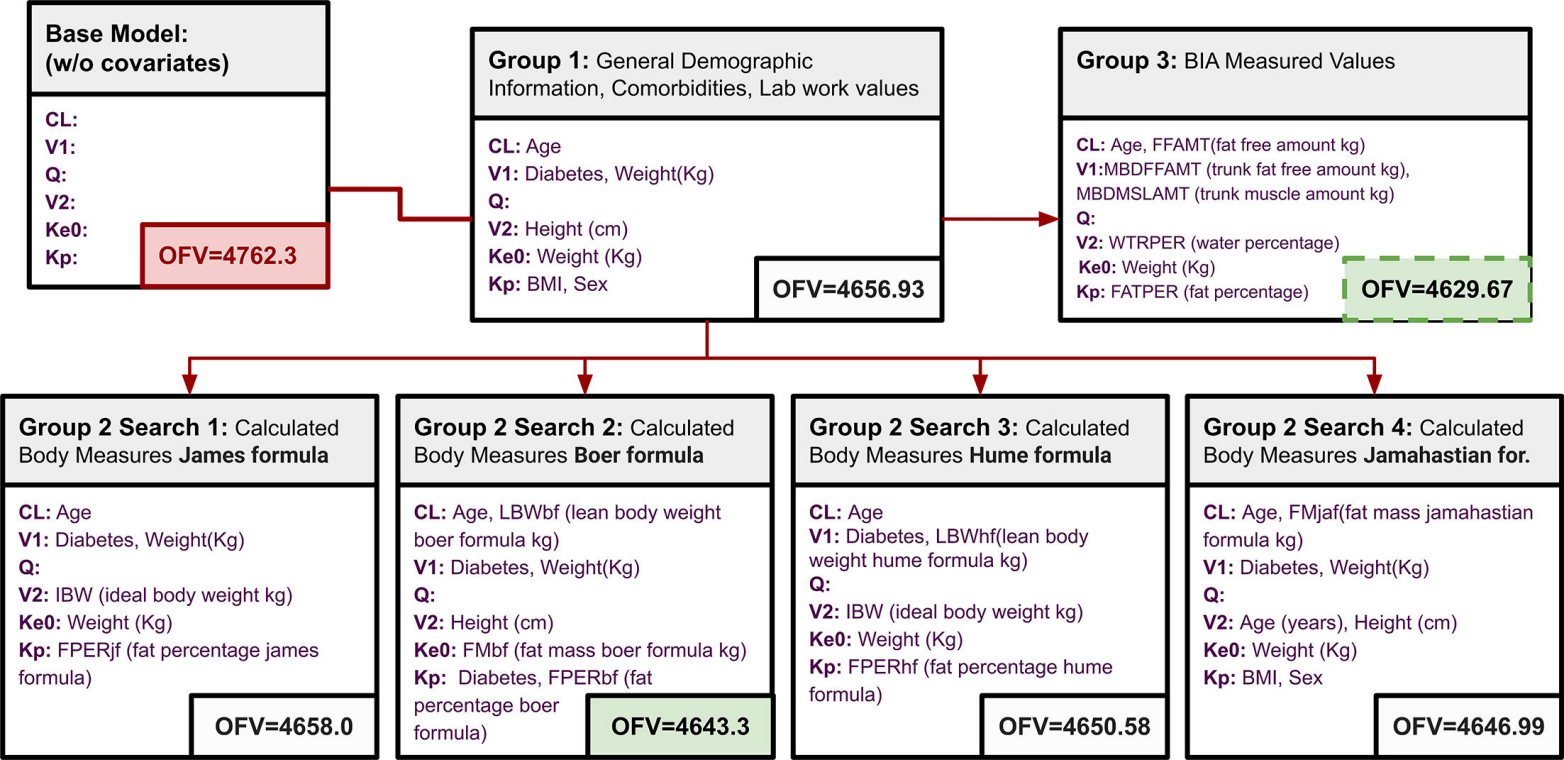
Stepwise covariate modeling selection of standard, calculated, and measured body metrics on biophase popPK model of total cefazolin plasma and subcutaneous adipose tissue exposure.

The initial structural model was focused on modeling cefazolin plasma concentrations alone. A two-compartment model provided the best description of plasma data. At the next stage simultaneously modeling both cefazolin plasma and subcutaneous adipose tissue concentrations was performed. A two-compartment systemic disposition model with a modified biophase compartment provided the best description of plasma and subcutaneous adipose tissue concentrations. A model schematic of the final structural model with differential equations is depicted in Fig. 4. Briefly, the final popPK model was parameterized by clearance (CL), central compartment volume (*V*_1_), intercompartmental clearance (*Q*), and peripheral compartment volume (*V*_2_), equilibrium rate constant between plasma and biophase compartment (*K*_e0_), plasma to biophase partition coefficient (*K*_p_). The final plasma base model incorporated random effects on all parameters including correlation between clearance (CL) and central compartment volume of distribution (*V*_1_). The correlation between CL and *V*_1_ was [*r* = 0.59], indicating moderate positive correlation between these pharmacokinetic parameters. A combined additive and proportional residual error model (combine 2) was selected to describe residual unexplained variability in the observed cefazolin plasma concentration data compared to either the additive or proportional models alone. A constant residual error model with lognormal distribution was selected to describe the unexplained residual variability in the biophase compartment. Inter-individual variability (IIV) was included for plasma parameters, CL, *V*_1_, and *V*_2_, modeled using normal distributions. IIV was included for the biophase parameters *K*_e0_, *K*_p_, modeled using lognormal distributions. The biophase structural model had an OFV value of 4,762.3 (Fig. 5) and provided a superior fit to all other iterations of plasma-subcutaneous co-model exploration. This model structure was then selected for further stepwise covariate modeling.

### Stepwise covariate modeling

A stepwise covariate modeling approach was conducted and structured into three sequential groups to systematically evaluate potential covariates. Figure 4 outlines the stepwise covariate modeling process across all groups. Initially, standard demographic parameters and laboratory values (Group 1) were assessed for their value in improving fits of the biophase popPK model. Age significantly influenced clearance (CL); the diagnosis of diabetes mellitus and body weight (kg) influenced of central compartment volume (*V*_1_); height (cm) influenced peripheral compartment volume (*V*_2_); body weight (kg) affected *K*_e0_; and both body mass index (BMI) and sex affected of *K*_p_. No covariates were identified on inter-compartmental clearance (*Q*). Inclusion of Group 1 covariates resulted in a substantial reduction of 105.37 points compared to the base biophase structural model.

Covariates selected from Group 1 SCM were available for evaluation alongside calculated body metrics (Group 2). In Group 2, calculated body metrics using the Boer’s formula (Search 2) provided the most substantial improvement over all other formulas, yielding an OFV reduction of 119 points from the biophase structural model. Briefly, age and lean body weight (kg) significantly influenced clearance (CL); the diagnosis of diabetes mellitus and body weight (kg) influenced central compartment volume (*V*_1_); height (cm) influenced peripheral compartment volume (*V*_2_); fat mass (kg) affected *K*_e0_; and both diagnosis of diabetes mellitus and body fat percentage (kg) influenced *K*_p_. No covariates significantly impacted inter-compartmental clearance (*Q*).

Finally, covariates selected from Group 1 were available for selection alongside BIA-derived body composition measurements (Group 3). The covariate search conducted with BIA-derived measures (Group 3) achieved the lowest OFV of all stepwise covariate model exploration, with 132.63 point reduction from the biophase structural model. Age and fat-free mass (kg) were selected as covariates affecting CL, trunk fat-free mass (kg) and trunk muscle mass (kg) as covariates affecting *V*_1_, body water percentage for *V*_2_, body weight (kg) for *K*_e0_, and body fat percentage for *K*_p_. No covariates were selected for *Q*.

Figure 5 further illustrates the selected covariates from SCM according to each group and sub-search. The final model was determined to be a two-compartment biophase model with covariates of measured body composition (Group 3). The population estimates for the final popPK model were 3.9 (L/h), 3.6 (L), 11.0 (L/h), 1.1 (L), 1.91 (h⁻¹), and 0.37, for CL, *V*_1_, *Q*, *V*_2_, and *K*_eo_, *K*_p_, respectively (Table S2). Model diagnostic plots are provided in Fig. S1 to S4. Non-parametric bootstrap analysis confirmed the model’s stability, with median bootstrap estimates closely mirroring the point estimates, and a convergence rate of 99.8% (Table S2).

## DISCUSSION

Throughout the course of this study, several observational and methodological insights came to light. For example, the recruitment of normal-weight participants proved more difficult than anticipated (*n* = 10), which led to the recruitment of overweight subjects (BMI = 25–29.9) to represent the non-obese subgroup for PK analysis. The difficulties of recruiting non-obese subjects may indicate a shift toward increasing levels of obesity in the United States (53). Another peculiar visual trend is that adiposity, as defined by BMI and body fat percentage, seems inversely proportional to age in our study population (Table 1). This observation aligns with CDC reports of severe obesity emerging at increasingly younger ages (54). Careful methodological considerations were taken to maintain the integrity of subcutaneous adipose tissue samples. Although electrocautery promotes hemostasis during the course of tissue collection, samples are repeatedly exposed to extreme temperatures. Due to the concerns of both volume loss and degradation of cefazolin during sample collection, our surgeons excised all subcutaneous adipose samples with a cold scalpel. The labor-intensive precautions taken were necessary to ensure accurate reporting of cefazolin concentrations that represent true in-vivo exposure instead of tissue handling artifacts.

When observing plasma concentrations alone, subjects ≥120 kg receiving 3 g vs <120 kg receiving 2 g have similar drug exposure across BMI strata, with only a slight deviation for BMI ≥40. These findings highlight that the institutional standard weight threshold for dosing is adequate for plasma drug exposure. Further analysis showed a significant increase in clearance among subjects ≥120 kg vs <120 kg, in line with prior publications (4.35 vs 3.2 L•h⁻¹), as well as volume of distribution (13.56 vs 11.07 L) (23, 55–57). Therefore, it appears that both volume of distribution and clearance increase in relation to total body weight. The disparity in volume of distribution is also seen in subjects with BMI > 40 when receiving 2 g vs 3 g doses (9.44 vs 13.19 L). This suggests that cefazolin may be partitioning differently between plasma and peripheral tissues with increasing body weight and is likely due to differences in body volumes between groups (i.e., body composition). With proportionately higher clearance and volume of distribution in the 3 g vs 2 g dose group, the terminal half-life remained unchanged (2.66 vs 2.44 h). Although, a dose increase from 2 g to 3 g compensates for the augmented clearance by maintaining a comparable total plasma AUC of cefazolin (Fig. 2), the dose‐normalized AUC (0.36 vs 0.27 h•L⁻¹) is reduced because of this increased clearance capacity. While it appears current weight-based dosing guidelines for cefazolin provide adequate plasma exposure, it is crucial to recognize that, in the context of surgical prophylaxis, the drug’s site of action resides within target tissues.

When observing subcutaneous adipose tissue concentrations alone, it was evident that therapeutic cefazolin exposure at the site of action was not achieved (Fig. 3). Of all subjects only 40.2% remained above an MIC 4 µg/mL, 17.2% above MIC 8 µg/mL, and 3.4% above MIC 16 µg/mL for the duration of surgery. Despite this alarmingly low target attainment rate, there were clear apparent trends of decreasing percentage of target attainment with increasing BMI and fat percentage across all MIC thresholds (Table 3). A plausible explanation for this inadequate percentage of target attainment is the prevalence of Class 1–3 obesity among ~80% of enrolled subjects, leading to increased adiposity and reduced cefazolin distribution into subcutaneous adipose tissues. Similar subcutaneous adipose tissue concentration ranges were previously reported by Chen et al. (58), in an obese population (however that study used a lower MIC target of 1 µg/mL) (58). Despite a dose escalation of 2 g to 3 g for subjects weighing more than 120 kg, there was still a marked lower percentage of target attainment, highlighting the limitations of weight-based dosing in achieving sufficient subcutaneous adipose tissue exposure. Therefore, for drugs that exert their effects in tissues of individuals with obesity, it is critical to evaluate PK beyond plasma concentrations alone.

It is important to note the exposure level necessary to prevent SSIs remains unclear. Due to the absence of a consensus on optimal MIC targets, with published values ranging from 0.5 to 32 µg/mL, our analysis evaluated multiple MIC thresholds. Considering an approximate 20% unbound fraction of cefazolin in plasma (59–63), a total tissue concentration of 8 µg/mL translates to an unbound MIC of roughly 1.6 µg/mL. This MIC target aligns closely with the epidemiological cutoff for cefazolin against Staphylococcus aureus, the most common pathogen implicated in SSIs (64, 65). The unbound fraction of cefazolin in plasma, although well supported in literature, is yet to be investigated in adipose tissues. As with all beta-lactams, Cefazolin exhibits time-dependent antimicrobial activity, characterized by time above the MIC. Yet in a surgical prophylaxis setting 100% time above MIC is a widely accepted target (3, 51, 52). Therefore, values of target attainment are simply reported subcutaneous adipose concentrations at the beginning and end of surgical duration. Further research is necessary to ensure the predictive performance of tissue exposure past the surgical duration.

Microdialysis has been used to report unbound ISF concentrations of respective tissues (66). Contamination from venous or arterial blood by damaged vessels from tunneled microdialysis probes is seldom considered a source of error in reported ISF concentrations. Yet, in a surgical setting where patients are administered anticoagulants, the continual flow of dialysate fluid may prevent blood clotting and may artificially increase reported ISF concentrations. Moreover, due to the added infection risk of introducing microdialysis probes subcutaneously, this approach was excluded from our study design. Cefazolin concentrations in plasma as well as subcutaneous adipose were comparable to previously reported concentrations although reported interstitial fluid (ISF) concentrations of the subcutaneous adipose were marginally higher (23, 56, 67, 68). Nevertheless, a significant strength of our study is the rigorous quantification of tissue concentrations using a fully validated LC-MS/MS method (48).

Our study sought to develop a robust popPK model of cefazolin that would directly compare standard anthropometric values, calculated body composition values, and measured body-composition values, with the overarching goal of identifying which set of parameters most accurately characterized variability in both plasma and subcutaneous adipose drug exposure. Our final popPK model incorporating measured body composition parameters demonstrated superior performance compared to other models tested in the SCM process. This is evidenced by the lowest OFV, reduced inter-individual variability (IIV), and improved overall fit. Within this final model, age and total fat-free mass (FFAMT) were identified as key covariates influencing cefazolin clearance (CL). Previous studies identified age, BMI, creatinine clearance, and total body weight as significant predictors of clearance (33, 46, 55, 69). However, despite the availability of these traditional covariates for selection, our analysis showed that age and FFAMT were more strongly correlated. Therefore, it seems measures of lean body weight provide superior predictive capability compared to total body weight alone. Further analysis revealed that abdominal fat-free mass (MBDFFAMT), serving as a surrogate marker of abdominal lean body weight, and muscle mass (MBDMSLAMT) significantly influenced the volume of distribution in the central compartment (*V*_1_). These findings are physiologically plausible as measures of abdominal lean body weight primarily capture intra-abdominal organ weight, which is closely associated with highly perfused compartments. This selection is also physiochemically relevant to water-soluble drugs like cefazolin, compared to total body weight, which includes adipose tissue with low water content. In the same context, total body water percentage (WTRPER) was significantly associated with the peripheral compartment volume (*V*_2_). Given cefazolin’s hydrophilic nature, this observation also aligns with physiological expectations. Individuals with higher total body water can exhibit increased distribution into peripheral tissues. Moreover, measured body fat percentage (FATPER) significantly influenced cefazolin partition into subcutaneous adipose tissue (*K*_p_). The rate constant for cefazolin transfer from the central compartment to subcutaneous adipose (*K*_e0_) notably decreased with increasing total body weight. These findings corroborate previous reports indicating decreased antibiotic distribution into subcutaneous adipose tissue among individuals with obesity (23, 25, 70).

In the absence of a BIA device to measure body composition, we have further explored calculated body composition values as covariates on our popPK model. The Boer formula proved to be the most significant LBW formula for covariate selection when evaluated alongside standard anthropometric measures. For a complete list of selected covariates using the Boer formula (Group 2), refer to Fig. 5. Briefly, age together with LBW proved to be influential covariates affecting the clearance of cefazolin, in agreement with our BIA-informed final PK model. The rate of drug transfer from plasma into subcutaneous adipose tissue (*K*_e0_) was dependent on fat mass, while fat percentage influenced cefazolin partition from plasma into subcutaneous adipose tissue (*K*_p_), a finding also in agreement with our BIA-informed final PK model. Therefore, calculated body composition parameters, particularly those obtained from the Boer formula, can act as refined clinical predictors for cefazolin exposure.

Overall, incorporating total body weight (TBW) improved our base biophase PopPK model; however, its selection may partly reflect institutional dosing practices rather than physiological relevance. Nevertheless, TBW remains a practical covariate for dose adjustments in plasma exposure. Still, body composition parameters appear significantly more predictive for achieving adequate cefazolin exposure in subcutaneous adipose tissues. Given these findings, consideration of body composition-driven dosing guidelines is warranted. Our popPK model suggests that individuals exceeding specific BIA thresholds (to be defined) may require higher cefazolin doses to achieve effective prophylactic exposure. A better understanding of the mechanisms of effect of body composition on cefazolin PK may help identify patients who require dose adjustment rather than relying on arbitrary weight thresholds. Replication of our findings, the feasibility of dosing on this new metric, and prospective comparative studies are needed before changes in surgical prophylaxis practice can be expected.

### Conclusion

In conclusion, although plasma exposure of cefazolin was adequate, utilizing the current institutional dosing regimen, subcutaneous adipose tissue exhibited significantly lower concentrations often falling below MIC thresholds during surgery. It should also be noted that cefazolin exposure in plasma does not accurately reflect exposure at the site of action. Therefore, greater attention should be given to prioritize measuring drug concentrations specifically at the site of action. Lastly, the inclusion of measured body composition metrics offers superior insight into the variability of cefazolin exposure across diverse patient populations, as compared to standard anthropometric values.

## SUPPLEMENTAL MATERIAL

Fig. S1 aac.01677-25-s0001.tiff VPC of popPK model of total cefazolin plasma exposure after intravenous administration of 2 or 3g dose in subjects receiving abdominal surgery.

Fig. S2 aac.01677-25-s0002.tiff Observed vs predicted plots of popPK model of total cefazolin plasma exposure after intravenous administration of 2 or 3g dose in subjects receiving abdominal surgery.

Fig. S3 aac.01677-25-s0003.tiff Normalized prediction distribution error plots of total cefazolin plasma exposure after intravenous administration of 2 or 3g dose in subjects receiving abdominal surgery.

Fig. S4 aac.01677-25-s0004.tiff Standard fit of biophase popPK model of total cefazolin subcutaneous adipose tissue exposure after intravenous administration of 2 or 3g dose in subjects receiving abdominal surgery.

Supplemental material aac.01677-25-s0005.docx Supplemental figure legends.

Table S1 aac.01677-25-s0006.docx Comparison of standard, calculated, and measured body values.

Table S2 aac.01677-25-s0007.docx Final population pharmacokinetic model parameters of total cefazolin based on both plasma and subcutaneous tissue concentrations.

ASM does not own the copyrights to Supplemental Material that may be linked to, or accessed through, an article. The authors have granted ASM a non-exclusive, world-wide license to publish the Supplemental Material files. Please contact the corresponding author directly for reuse.
