# Supplementary material for: Cefazolin surgical prophylaxis in obesity: a body composition-driven population pharmacokinetic approach
Source: Antimicrob Agents Chemother. 2026 May 29;70(7):e01677-25. doi: 10.1128/aac.01677-25 (PMC13321816; doi:10.1128/aac.01677-25)
Supplement: Supplemental material — Supplemental figure legends. [file aac.01677-25-s0005.docx]

**SUPPLEMENTARY FIGURE LEGENDS:**

- **Supplemental Figure 1: Abbreviations:** Cp is the observed concentration in plasma of subjects**;** VPC is the prediction-corrected visual predictive check of the pharmacokinetic model. The 5th, 50th, and 95th percentiles of the simulated cefazolin concentrations are plotted over time, with the observed data overlaid. The blue and pink shaded areas represent the confidence intervals around the prediction intervals (dashed lines).
- **Supplemental Figure 2: Legend:** Goodness-of-fit plots**;** The black line indicates the line of identity. Blue circles represent the observed concentrations plotted against the corresponding predicted concentrations. The yellow line shows the trend. **Left panel:** Observed plasma concentrations (µg/mL) versus population predicted concentrations (PRED), which do not account for individual variability. **Right panel:** Observed plasma concentrations (µg/mL) versus individual predicted concentrations (IPRED), which include individual variability.
- **Supplemental Figure 3: Abbreviations:** Goodness of fit plots; The black line represents the line of identity; NPDE, is the normalized prediction distribution errors
- **Supplemental Figure 4: Legend:** Goodness-of-fit plots**;** The solid green line indicates the population fit. The purple line indicates the individual fit. The dashed green line indicates the BIA adjusted fit after covariate inclusion. Blue circles represent the observed subcutaneous tissue concentrations. **Black panel:** Standard fit of the first 12 subjects’ subcutaneous cefazolin subcutaneous adipose tissue concentration (µg/mL). **Red panel:** Selection of subjects with poorest fit of subcutaneous cefazolin tissue concentration (µg/mL).
