# Supplementary material for: Cefazolin surgical prophylaxis in obesity: a body composition-driven population pharmacokinetic approach
Source: Antimicrob Agents Chemother. 2026 May 29;70(7):e01677-25. doi: 10.1128/aac.01677-25 (PMC13321816; doi:10.1128/aac.01677-25)
Supplement: Table S1 — Comparison of standard, calculated, and measured body values. [file aac.01677-25-s0006.docx]

| Supplement Table 1: Comparison of Standard, Calculated, and Measured Body Values | | | | | | |
| --- | --- | --- | --- | --- | --- | --- |
| Characteristics | ALL | | Dosage | | | |
|  |  | | **2g** | | **3g*** | |
| Standard Body Values | Mean (SD) | Median [Range] | Mean (SD) | Median [Range] | Mean (SD) | Median [Range] |
| Height (cm) | 172.2 (13.01) | 172.26 [63.5] | 170.06 (11.9) | 170.1 [63.5] | 176.74 (14.33) | 173.9 [55.8] |
| Weight (Kg) | 112.9 (31.7) | 112.9 [162.5] | 95.4 (18.3) | 98.6 [64.5] | 146.9 (24.6) | 142.9 [97.4] |
| BMI (kg/m^2^) | 38 (9.72) | 38.03 [49.2] | 33.2 (6.96) | 33.5 [29.2] | 47.3 (7.48) | 46.2 [37.5] |
| Calculated Body Metrics | Mean (SD) | Median [Range] | Mean (SD) | Median [Range] | Mean (SD) | Median [Range] |
| IBW (kg) | 67.41 (11.49) | 65.7 [56.6] | 65.63 (10.6) | 65.7 [56.6] | 71.01 (12.65) | 68 [49.7] |
| LBWjaf (kg) | 79.8 (14.15) | 78.88 [68.59] | 79.14 (15.56) | 77.77 [68.59] | 81.06 (11.31) | 82.52 [48.68] |
| LBWjf (kg) | 59.48 (15.33) | 55.91 [66.28] | 57.9 (12.01) | 55.03 [50.68] | 62.71 (20.41) | 56.88 [66.28] |
| LBWbf (kg) | 66.47 (16.45) | 62.06 [83.88] | 59.68 (10.81) | 58.03 [45.05] | 80 (17.68) | 76.09 [69.84] |
| LBWhf (kg) | 63.69 (13.61) | 62.21 [72.84] | 57.36 (8.99) | 57.79 [41.54] | 76.19 (12.81) | 74.17 [55.68] |
| FMjaf (kg) | 33.07 (33.63) | 34.94 [183.14] | 16.62 (26.35) | 25.04 [104.42] | 65.91 (19.81) | 63.08 [89.51] |
| FMjf (kg) | 53.39 (30.4) | 46.73 [158.05] | 37.45 (16.43) | 35.81 [62.51] | 84.26 (28.06) | 77.25 [29.17] |
| FMbf (kg) | 46.47 (20.27) | 44.2 [90.22] | 35.72 (13.54) | 35.68 [50.59] | 66.97 (14.81) | 64.78 [56.62] |
| FMhf (kg) | 49.18 (20.32) | 47.61 [102.98] | 38.04 (12.48) | 39.53 [47.08] | 70.77 (14.78) | 66.62 [65.28] |
| Measured Body Values | Mean (SD) | Median [Range] | Mean (SD) | Median [Range] | Mean (SD) | Median [Range] |
| *Whole Body Values* |  | |  | |  | |
| FATAMT (kg) | 44.4 (20.3) | 43.2 [104.1] | 36.1 (16.3) | 34.5 [63] | 73.8 (18.4) | 56.1 [84.3] |
| FATPER (%) | 37.94 (10) | 40.2 [40.2] | 34.9 (10.33) | 35.2 [40.2] | 43.6 (6.43) | 44.5 [25] |
| MSLAMT (kg) | 65.07 (16) | 61.8 [79.7] | 60.6 (12.8) | 59.7 [61] | 73.8 (18.42) | 74.1 [68.9] |
| FFMAMT (kg) | 68.47 (16.77) | 65 [83.5] | 34.97 (10.33) | 62.8 [63.9] | 43.6 (6.43) | 78 [72.2] |
| *Abdominal Trunk Values* |  | |  | |  | |
| MBDFATAMT (kg) | 21.9 (10) | 21.8 [59.1] | 17.4 (6.11) | 18.2 [24.1] | 30.86 (10.45) | 29.1 [54.2] |
| MBDFATPER (%) | 35.68 (9.1) | 37.1 [44.1] | 33.01 (8) | 35.1 [39] | 40.9 (8.05) | 41.7 [39.1] |
| MBDFFMAMT (kg) | 37.33 (7.38) | 37.1 [36.1] | 34.39 (6.44) | 34.3 [35] | 42.98 (5.78) | 43.1 [24.8] |
| MBDMSLAMT (kg) | 35.67 (7.15) | 25.4 [34.9] | 32.82 (6.21) | 32.7 [33.7] | 41.18 (5.61) | 41.2 [24.1] |
| *Body Water Values* |  | |  | |  | |
| WTRAMT (kg) | 48.92 (12.88) | 46.7 [67.3] | 45.1 (9.6) | 44.7 [45.4] | 56.4 (15.34) | 55.5 [59.3] |
| WTRPER (%) | 44.1 (6.69) | 42.7 [28.2] | 45.9 [6.8] | 45.3 [28.2] | 40.57 (4.96) | 40.2 [18.6] |
| ECW (kg) | 21.6 (4.75) | 21.4 [25] | 19.25 (3.02) | 19.3 [13.9] | 26.4 (3.99) | 26.3 [16.5] |
| ECWPER (%) | 44.8 (3.2) | 45 [14.5] | 44.7 (3.02) | 45.3 [13.4] | 44.8 (3.6) | 43.9 [13.4] |
| ICW (kg) | 27.25 (8.4) | 25 [42.9] | 24.17 (6.24) | 23.3 [32.1] | 33.3 (9.07) | 31.1 [35.3] |
| Abbreviations: BMI=body mass index; IBW=ideal body weight; LBWjaf=lean body weight according to Janmahasatian formula; LBWjf=lean body weight according to James formula; LBWbf=lean body weight according to Boer formula; LBWhf=lean body weight according to Hume formula; FMjaf=fat mass according to Janmahasatian formula; FMjf=fat mass according to James formula; FMbf=fat mass according to Boer formula; FMhf=fat mass according to Hume formula; FATAMT=measured total body fat mass; FATPER=measured total body fat percentage; MSLAMT=measured total body muscle mass; FFMAMT=measured total body fat free mass; MBDFATAMT=measured abdominal fat mass; MBDFATPER=measured abdominal fat percentage; MBDMSLAMT=measured abdominal muscle mass; MBDFFMAMT=measured abdominal fat free mass; WTRAMT=measured total body water mass; WTRPER=measured total body water percentage; ECW=measured total body extracellular water; ECWPER=measured total body extracellular water percentage; ICW= measured total body intracellular water.  *One subject in the 3 gram group weighed less than 120 kg (118 kg). | | | | | | |
