# Supplementary material for: Cefazolin surgical prophylaxis in obesity: a body composition-driven population pharmacokinetic approach
Source: Antimicrob Agents Chemother. 2026 May 29;70(7):e01677-25. doi: 10.1128/aac.01677-25 (PMC13321816; doi:10.1128/aac.01677-25)
Supplement: Table S2 — Final population pharmacokinetic model parameters of total cefazolin based on both plasma and subcutaneous tissue concentrations. [file aac.01677-25-s0007.docx]

| **Supplementary Table 2**: Final population pharmacokinetic model parameters of total cefazolin based on both plasma and subcutaneous tissue concentrations | | | | | | |
| --- | --- | --- | --- | --- | --- | --- |
| **Parameter** | **Value** | | **Stoch. Approx.** | | **Cond. Mode** | **Bootstrapped Median (95% CI)**^a,b^ |
|  |  |  | **S.E.** | **R.S.E.(%)** | **Shrinkage (%)** |  |
| **Fixed Effects** | | | | | |  |
| **CL_pop_ (L/h)** | 3.9 | | 0.78 | 20.25 | 8.63 | 3.7 (2.6-5.3) |
| **𝛃**_CL-AGE_ | -0.011 | | 0.0023 | 20.49 |  | -0.01 (-0.015, -0.0058) |
| **𝛃**_CL-FFMAMT_ | 0.0052 | | 0.0021 | 39.8 |  | 0.0055 (0.002-0.0089) |
| **V1_pop_ (L)** | 3.6 | | 0.77 | 21.48 | 10.97 | 3.5 (2.2-5.6) |
| **𝛃**_V1-MBDFFMAMT_ | -0.64 | | 0.26 | 41.01 |  | -0.72 (-1.37, -0.13) |
| **𝛃**_V1-MBDMSLAMT_ | 0.69 | | 0.27 | 39.46 |  | 0.77 (0.15-1.44) |
| **Q_pop_ (L/h)** | 11 | | 1.2 | 10.33 | 46.85 | 12 (9.6-17) |
| **V2_pop_ (L)** | 1.1 | | 0.45 | 40.53 | 25.57 | 1.3 (0.59-2.9) |
| **𝛃**_V2-WTRPER_ | 0.033 | | 0.0081 | 24.3 |  | 0.029 (0.012-0.047) |
| **ke0_pop_ (h^-1^)** | 1.91 | | 1.75 | 91.49 | 54.51 | 2 (0.26-14.53) |
| **𝛃**_ke0-WTKG_ | 0.025 | | 0.0057 | 22.59 |  | 0.025 (0.0087-0.045) |
| **Kp_pop_** | 0.37 | | 0.12 | 32.67 | 27.6 | 0.43 (0.26-0.68) |
| **𝛃**_Kp-FATPER_ | -0.063 | | 0.0077 | 12.31 |  | -0.066 (-0.077, -0.053) |
| **Standard Deviation of the Random Effects** | | | | | | |
|  | **Value** | **C.V.(%)** |  | | |  |
| **Ω_CL_** | 0.27 | 26.98 | 0.027 | 10.1 |  | 26% (20-32%) |
| **Ω_V1_** | 0.25 | 25.47 | 0.026 | 10.29 |  | 25% (18-31%) |
| **Ω_Q_** | 0.41 | 43.21 | 0.081 | 19.52 |  | 41% (17-69%) |
| **Ω_V2_** | 0.33 | 33.65 | 0.05 | 15.3 |  | 33% (20-45%) |
| **Ω_ke0_** | 0.68 | 77.37 | 0.3 | 44.3 |  | 89% (39-151%) |
| **Ω_Kp_** | 0.51 | 54.55 | 0.11 | 21.09 |  | 54% (25-73%) |
| **Correlations** | | | | | | |
| **correlation V1 & CL** | 0.59 | | 0.1 | 16.78 |  | 0.6 (0.33-0.84) |
| **Error Model Parameters** | | | | | | |
| **𝜎_additive_** | 51.4µg/mL | | 11 | 21.38 |  | 52.3µg/mL (9.8-87.4µg/mL) |
| **𝜎_proportional_** | 9.5% | | 0.79 | 8.38 |  | 8.9% (4.9-12%) |
| **𝜎_constant_** | 6.1µg/mL | | 1.1 | 17.37 |  | 5.4µg/mL (3.5-7.3µg/mL) |
| **Abbreviations**: β, coefficient on the specified PK parameter-covariate (subscripted) weighted to the median; Ω, random effect variance for each parameter;𝜎_additive_, plasma additive error model parameter; 𝜎_proportional,_ plasma proportional error model parameter; 𝜎_constant,_ subcutaneous biophase error model parameter; CL, clearance; V1, plasma volume of distribution; Q, intercompartmental clearance; V2, peripheral tissue volume of distribution; Ke0, rate constant of equilibrium between plasma compartment and biophase; Kp, plasma to subcutaneous fat partitioning coefficient; R.S.E, relative standard error; S.E, standard error.  ^a^95% CI was estimated from 1000 resampled datasets using the final population pharmacokinetic model.  ^b^The convergence rate in bootstrapping was 99.8%. | | | | | | |
